# Bilateral passive thermal management for dynamical temperature regulation

**DOI:** 10.1038/s41598-024-53433-1

**Published:** 2024-02-04

**Authors:** Bingyao Li, Sicheng Zeng

**Affiliations:** 1 Shanghai University of Finance and Economics, Yangpu, Shanghai, 200433 China; 2 Shanghai Business School, Fengxian, Shanghai, 200235 China; 3 Department of Material Science and Engineering, Southern University of Science and Technology, Nanshan, Shenzhen, 518055 China

**Keywords:** Energy science and technology, Engineering

## Abstract

Space heating and cooling of buildings account for $\sim$ 30% of energy consumed globally. As a result, efforts to reduce the carbon footprint associated with building temperature regulation are crucial to achieving sustainability goals. Passive radiative cooling and sun heating are two promising innovations that can help reduce the energy consumption associated with building temperature regulation. However, these methods are constrained by factors such as climate zones and seasonal changes. In this work, we demonstrate numerically that bilateral photonic metamaterials enabled by vanadium dioxide (VO_2_) nanoparticles embedded into polyethylene nanogratings can achieve dynamically modulate solar absorptance at one side in winter and achieve radiative cooling on another side. In cold weather, the solar absorptance of this metastructure can change from 0.93 to 0.2 at a critical temperature when VO_2_ changes from a metallic to an insulating state. This metastructure consists of broadband transparent polyethylene (PE) gratings on the reflective silver (Ag) gratings with the same period and filling ratio. These gratings are deposited on top of the Polydimethylsiloxane (PDMS) thin film. The VO_2_ nanoparticles enable dynamic solar absorptance modulation for winter space heating, while the PDMS with strong infrared emittance derived from molecular vibrations over the atmospheric window for summer radiative cooling. Temperature response simulations validate that this bilateral design achieves   6 °C subambient temperature drop at noontime in the summer and stabilizes its temperature around 22 °C during the daytime in the winter. This passive dynamical thermal regulation technique can be deployed for energy-saving targets of buildings, vehicles, and greenhouses in areas with large temperature fluctuations.

## Introduction

Buildings make a significant contribution to global energy consumption, accounting for 30% of annual energy usage, and they also generate 10% of global greenhouse gas^1–4^. This presents substantial environmental and economic challenges in our pursuit of a sustainable future. Nearly half (48%) of this energy consumption is attributed to space heating and cooling, emphasizing the importance of innovative solutions for managing building temperatures and achieving net-zero-energy buildings^5–7^. Passive radiative cooling materials and structures can harness the cooling resource of cold outer space, providing an alternative approach to reducing air-conditioning energy usage, particularly during hot summers. Additionally, research is being conducted on low-emissivity (low-$\epsilon$) building envelopes that restrict radiative heat dissipation in colder regions, thereby reducing energy consumption for cooling and heating systems and lowering the carbon footprint associated with daily building operations^8^. However, considering various climate patterns on Earth, the static radiative properties of such methods are impeded by the variable and unpredictable nature of these weather conditions^9^. Moreover, the ever-increasing occurrence of extreme weather events such as heat and cold waves demands more intelligent and adaptable building envelope designs that can respond to diverse weather conditions^10–13^. Hence, a promising solution for net-zero-energy buildings requires the capability to dynamically adjust the radiative properties of buildings envelopes in response to weather conditions, which can enhance building energy efficiency and enable buildings to operate sustainably.

**Figure 1:**
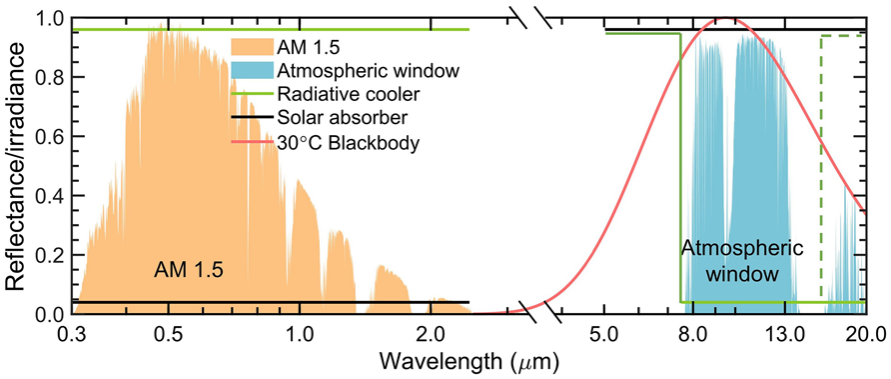
Photonic manipulation enabled temperature control. The spectral characteristics of the solar absorber (represented by solid black) and the radiative cooler (represented by solid or dashed green). The dashed green line represents a radiative cooler designed for subambient cooling, aiming to achieve an optimal reduction in temperature. In contrast, the solid greenhouse represents a radiative cooler designed for above-ambient radiative cooling, with the goal of maximizing radiative cooling power. The ASTM standard solar irradiance spectrum is depicted by the orange area, the mid-infrared atmospheric window is the blue area, and the solid red line represents the normalized spectrum of blackbody irradiance at 30 °C (shown in solid red).

To achieve this objective, we can utilize two boundless sources: the Sun, (5800 K), which can serve as the heat source to provide energy, while outer space (3 K) acts as the cold source to receive thermal energy from the Earth radiatively. These sources have the potential to fulfill the heating and cooling requirements of buildings without relying on fossil fuels^14^. For optimal passive daytime radiative cooling, materials should possess high reflectance across solar wavelengths ranging from 0.3 to 2.5 μm while showing a high thermal emittance approaching unity over the first atmospheric window (8–13 μm) for subambient radiative cooling purpose (represented by the dashed green line in Fig. 1) or over both the first and second transparent window (8–13 μm and 16–21 μm) for above-ambient cooling (solid green line)^5^. Conversely, the ideal selective solar absorber should own high solar absorptance to effectively harvest solar energy for heating effect and relatively low thermal emittance to minimize the radiative heat release through surface thermal emission 1).

Significant advancements in materials science, photonics, plasmonics, and heat transfer have led to profound insights and understanding, driving notable technological advancements in the field of solar heating and radiative cooling^15–20^. While impressive spectral selectivity has been demonstrated in the aforementioned studies, statically tuned devices can only provide monofunctional energy reduction by either solar energy harvesting or radiative cooling, catering to specific climate zones. This can result in overheating during summers or overcooling in winters due to their fixed spectral characteristics, failing to meet the switchable cooling and heating requirements necessary for energy-saving goals across seasonal changes^6^. To address this challenge, a surface with dynamic tunability between heating and cooling is required, capable of adjusting based on external temperature or through mechanical/electrical stimuli, providing switchable modes suitable for different climate zones. To achieve this, a surface that can modulate its reflectance according to a critical temperature ($T_c$) change is highly demanded. Below the critical temperature ($T_{emitter}$ < $T_c$), the surface functions in solar heating mode, while above the critical temperature ($T_{emitter}$ > $T_c$), it operates in radiative cooling mode. This enables dynamic passive thermal regulation during winter conditions, while static passive radiative cooling surfaces are essential for energy conservation in summer. Therefore, a bilayer surface can be designed with the capability for dynamic switchable solar heating on one side, suited for winter applications, and passive radiative cooling on the other side, suitable for summer deployment.

In recent studies, researchers have explored the potential of vanadium dioxide (VO_2_) for achieving switchable radiative cooling, enabling dynamic passive thermal modulation. Kim et al. successfully achieved dynamically switchable radiative cooling by combining an emitter (VO_2_/silicon (Si)/silver (Ag)) with the film with high solar reflectance (a three-layered photonic crystal)^21^. Liu et al. proposed a grating pattern of VO_2_ on a PDMS substrate, which shows mechanical-induced tunable thermal emittance and the top nanoporous polyethylene (PE) films with high solar reflectance helps minimize the solar heating effect, creating an intelligent radiative cooling system capable of efficient thermal regulation^22^. While the phase-transition of pure VO_2_ happens at approximately 68 °C and it is not feasible for real-life applications since it is higher than regular air temperatures, the doping of VO_2_ with elements such as tungsten (W) or strontium (Sr) can lower this transition temperature to a temperature region closer to the ambient, making switchable radiative cooling of VO_2_ more feasible in practical applications. The photonic structures mentioned earlier concentrates on the spectral modulation of the mid-infrared wavelengths from 5 to 25 μm while the incorporation of a reflector with high solar reflectance is required to minimize heating effects.

This study presents a practical application of bilateral photonic metamaterials that polyethylene nanogratings contain VO_2_ nanoparticles. This metastructure enables the dynamic modulation of solar absorptance on one side, facilitating winter space heating while achieving radiative cooling on the other side. During cold weather, the VO_2_ undergoes a phase transition from metallic to insulating, decreasing solar absorptance from 0.93 to 0.2. The metastructure comprises transparent polyethylene gratings deposited on reflective silver gratings, both with the same period and filling ratio, on top of a Polydimethylsiloxane (PDMS) thin film. The VO_2_ nanoparticles enable dynamic solar absorptance modulation, while the PDMS facilitates radiative cooling in the summer due to its strong infrared emittance derived from molecular vibrations over the atmospheric window. We validate that this bilateral design can reach a subambient temperature drop of  6 °C at noontime in a typical summer weather condition and stabilizes its temperature around 22 °C during daytime in the winter. This passive dynamical thermal regulation technique has significant potential for energy-saving applications in buildings, vehicles, and greenhouses in areas with significant temperature fluctuations.

## Results

### The working mechanism and structure of the bilateral photonic metamaterials

**Figure 2:**
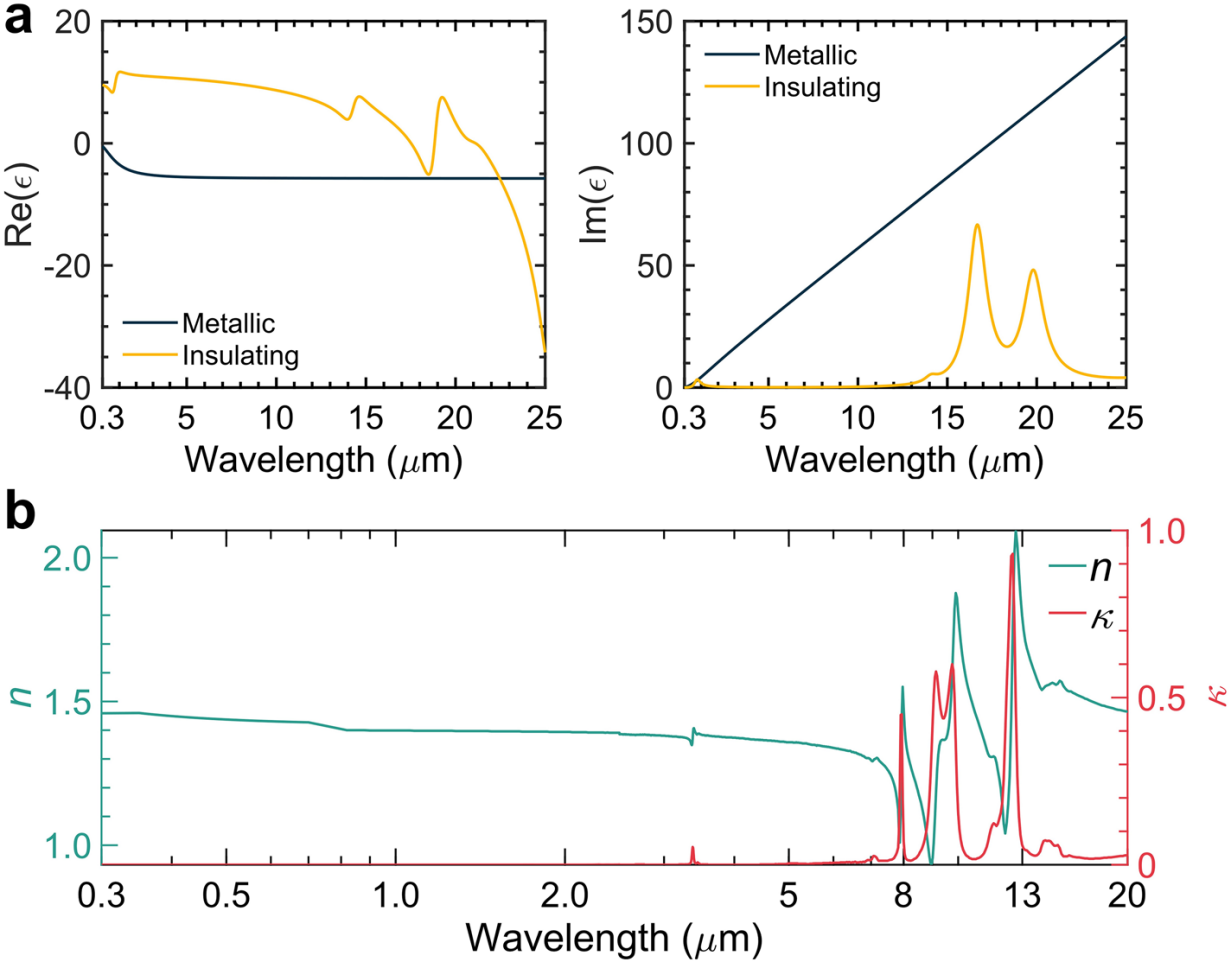
Optical feature of VO_2_ and PDMS. (**a**) Dielectric function of VO_2_ after co-doping by W and Sr^22^. (**b**) The complex refractive indices ($n + i\kappa$) of PDMS over wavelengths from 0.3 to 25 μm^23^.

According to a study conducted by Ono et al., VO_2_, a material that undergoes a solid-solid phase transition, demonstrates a reversible phase transition at a critical temperature, referred to as $T_c^{VO_2}$. Below this critical temperature, VO_2_ behaves as a semiconductor material with a narrow bandgap, allowing infrared light to pass through. Conversely, above $T_c^{VO_2}$, VO_2_ exhibits metallic properties, as indicated by its dielectric function shown in Fig. 2a. The switchable radiative response of VO_2_ nanoparticles has also been investigated, offering a scalable fabrication approach through rapid hydrothermal methods, which is advantageous compared to thin film deposition for VO_2_^24–26^. It is important to note that the phase transition of VO_2_ is not instantaneous, and the refractive index of VO_2_ exhibits a gradient within a constrained range, as observed in the trends ($T_c$ − $\Delta$*T*, $T_c$ $+$ $\Delta$*T*). Therefore, the dielectric function of VO_2_ over the phase-transition temperature region is defined as follows^22^:

1 $$\begin{aligned} \epsilon _{\text{ transition }}=\arctan \left( \frac{T-T_{\textrm{c}}}{\Delta T} \times 10\right) \times \frac{\epsilon _{\textrm{m}}-\epsilon _{\textrm{i}}}{2 \arctan 10}+\frac{\epsilon _{\textrm{m}}+\epsilon _{\textrm{i}}}{2} \end{aligned}$$

The permittivities of the metallic and insulating states are $\epsilon _m$ and $\epsilon _i$, respectively. Elements, including molybdenum (Mo) and tungsten (W) have been found to lower the phase-transition temperature ($T_c$) of VO_2_^22^. Goodenough (1971) proposed that the introduction of W atoms disrupts the V^4+^-V^4+^ pairs, which is important to the crystal structure when it is in the semiconducting phase. It is believed that the incorporation of metal elements also disrupts these homopolar V^4+^-V^4+^ bonds and induces the conversion of V^4+^ ions to V^3+^ ions. This destabilizes the semiconducting phase, leading to a lower temperature for the semiconductor-metal transition. To ensure realistic results, it is assumed that the permittivity of VO_2_ happens continuously at a narrow transition region [$T_c$ - $\Delta T$, $T_c$ + $\Delta T$]. In this particular study, the phase-transition temperature for doped VO_2_ was set to $T_c$ = 20 °C, with $\Delta T$ = 2 °C. The transition process of VO_2_ nanoparticles from an insulator to a metal can be switched reversibly within a short time ($\sim$ 100 fs)^27^. It is quick enough and has little effect on the performance in winter.

PDMS is a polymer that has been widely employed in the fabrication and prototyping of microfluidic chips^28^. It is a type of silicone elastomer that shows high transmittance over solar wavelengths, as depicted by the almost zero extinction coefficient ($\kappa$, solid red line in Fig. 2b). When its backside is deposited with a reflective metal thin film such as Ag or Aluminum (Al), it can serve as an efficient solar reflector to minimize solar heating. Apart from its unique optical property over solar wavelengths, PDMS shows high infrared thermal emittance from 8 to 13 μm due to molecular vibrations of chemical groups, as depicted in Fig. 2b. Specifically, the absorption peaks at 7.9 μm result from the CH_3_ symmetric bending in Si−CH_3_, while these two peaks at 9.3 μm and 9.8 μm are attributed to the vibration of Si−O−Si. The peak at 12.5 μm comes from the CH_3_ rocking in Si−CH_3_^29,30^. These absorption peaks overlap with the infrared transparent atmospheric window and render it a spectrally selective radiative cooler for efficient radiative cooling^7^.

**Figure 3:**
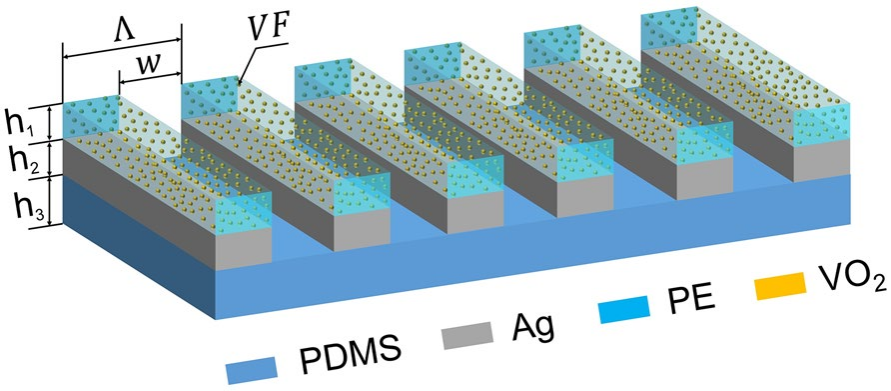
Schematic of the bilateral photonic metamaterials. The bottom layer is a PDMS thin film with a thickness ($h_3$) of 30 μm. The top gratings layer consists of stacked nanogratings of Ag ($h_2$) and PE ($h_1$) embedded with VO_2_ nanoparticles. The Ag and PE layers are 200 nm and 1 μm thick, respectively. The volume fraction (*VF*) and radius (*r*) of VO_2_ nanoparticles are 10% and 10 nm. The period ($\Lambda$) and filling ratio of the top nanogratings layer is 50 nm and 0.9, respectively.

**Figure 4:**
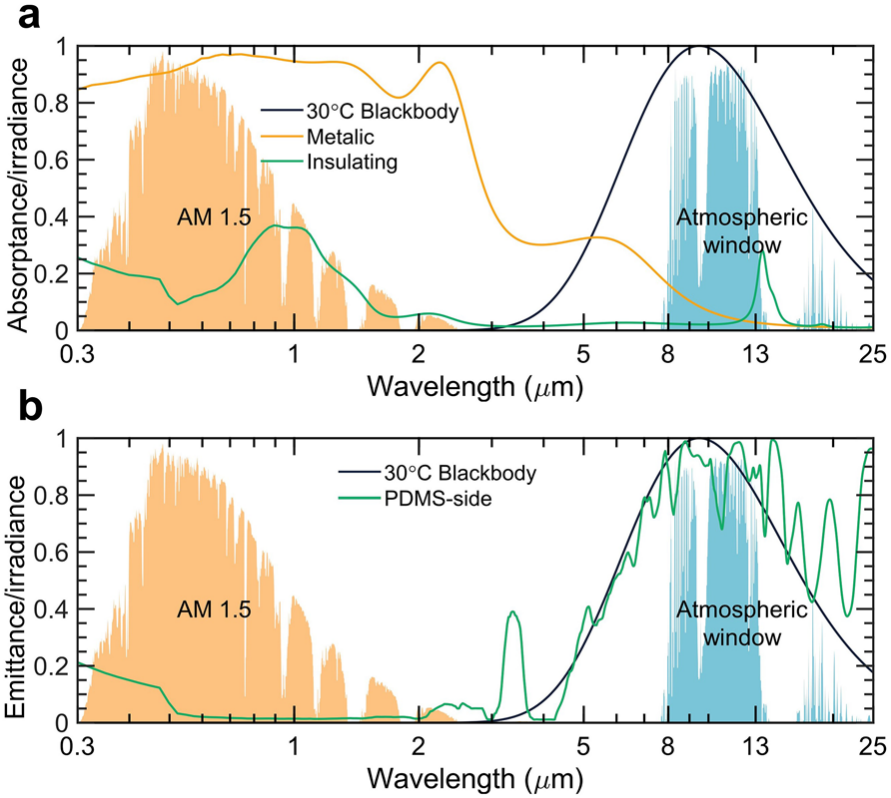
Spectrum of the bilateral photonic metamaterials. (**a**) Absorptance spectra of the bilateral metastructure when the nanograting layer faces the top at the metallic (solid orange line) and insulating (solid green line) states. (**b**) Emittance of the bilateral metastructure when the PDMS layer faces the top. The absorptance and emittance spectra are calculated with an incident angle of 0°.

By combining of unique optical properties of VO_2_ and PDMS, we propose a metastructure consisting of VO_2_ (thickness, $h_1$) and Ag (thickness, $h_2$) nanogratings over the PDMS thin film (thickness, $h_3$) from top to bottom, as depicted in Fig. 3. The period of stacked nanogratings and the width of the ridges are represented by the symbols $\Lambda$ and $\omega$, respectively. The filling ratio of the nanogratings is calculated by dividing the ridge width by the period, denoted by the symbol $\phi = \omega /\Lambda$. The volume fraction and radius of VO_2_ nanoparticles are denoted as *VF* and *r*. By employing the optimization tool in MATLAB to make the difference between solar absorptance largest, i.e., the solar absorptance of the proposed structure should approach 1 while VO_2_ in the metallic state, but its solar absorptance should approach 0 when VO_2_ is in the insulating state, the optimal configuration of the metastructure is: $h_1$ = 1 μm, $h_2$ = 200 nm, $h_3$ = 30 μm, *VF* = 10%, *r* = 10 nm, $\phi$ = 0.9. This metastructure can be utilized in either the nanograting layer or the PDMS layer facing the sky. The simulation process follows Alok et al.’s work^31^, and the dielectric function of Ag is extracted from previous literature^32^.

### Calculation of the overall solar absorptance and thermal emittance

The overall hemispherical solar reflectance, $\bar{R}$, is functions of wavelengths and incidents angles, which is expressed as:

2 $$\begin{aligned} \bar{R}(\lambda ,\theta , \phi )=\frac{\int _{0.3 \mu m}^{2.5 \mu m} I_{solar}(\lambda ,\theta , \phi ) R(\lambda ,\theta , \phi ) d \lambda }{\int _{0.3 \mu m}^{2.5 \mu m} I_{solar}(\lambda ,\theta , \phi ) d \lambda } \end{aligned}$$

where $\lambda$ is the wavelength of the solar radiation, $\phi$ is the azimuthal angle, and $\theta$ is the polar angle. $R(\lambda ,\theta , \phi )$ is the spectral directional reflectance.

The overall hemispherical thermal emittance, $\bar{\epsilon }$, is also a function of the wavelengths and incident angles, which is defined by:

3 $$\begin{aligned} \bar{\epsilon }(\lambda ,\theta , \phi )=\frac{\int _{2.5 \mu m}^{25 \mu m} I_{bb}(\lambda ,\theta , \phi )[1-R(\lambda ,\theta , \phi )] d \lambda }{\int _{2.5 \mu m}^{25 \mu m} I_{bb}(\lambda ,\theta , \phi ) d \lambda } \end{aligned}$$

where $I_{bb}(\lambda ,\theta , \phi )$ means the blackbody radiation intensity described by Planck’s law. $\bar{\epsilon }(\lambda ,\theta ,\phi )$ is the spectral directional absorptance at a certain operating temperature.

### Optical response of nanoparticles embedded into thin film

Clausius-Mossotti equation is used to express the effective dielectric function when VO_2_ nanoparticles are embedded in polyethylene^33,34^.

4 $$\begin{aligned} \epsilon _{eff}=\epsilon _{m}\left( \frac{R^{3}+2\alpha _{R} VF}{R^{3}-\alpha _R VF}\right) \end{aligned}$$

where $\epsilon _{m}$ is the matrix’s dielectric function, $\alpha _R$ is the electric dipole polarizability, and *R* and *VF* are the nanoparticles’ radius and volume fraction, respectively. Mie theory is an effective way to take doping nanoparticle size into account. Electric dipole polarizability is expressed using the Maxwell-Garnett formula and the Mie theory^35^,

5 $$\begin{aligned} \alpha _R=\frac{3jc^{3}}{2\omega ^{3}\epsilon _{m}^{3/2}}a_{1,R} \end{aligned}$$

where $a_{1,R}$ is the first electric Mie coefficient given by

6 $$\begin{aligned} a_{1,R}\!=\!\frac{\sqrt{\epsilon _{np}}\psi _{1}(x_{np})\psi _{1}^{'}(x_{m})\!-\!\sqrt{\epsilon _{m}}\psi _{1}(x_{m})\psi _{1}^{'}(x_{np}) }{\sqrt{\epsilon _{np}}\psi _{1}(x_{np})\xi _{1}^{'}(x_{m})\!-\!\sqrt{\epsilon _{m}}\xi _{1}(x_{m})\psi _{1}^{'}(x_{np})} \end{aligned}$$

where $\psi _{1}$ and $\xi _{1}$ are the first-order Riccati-Bessel functions expressed by $\psi _{1}(x)=xj_{1}(x)$ and $\xi _{1}(x)=xh_{1}^{(1)}(x)$ where $j_{1}$ and $h_{1}^{(1)}$ are first-order spherical Bessel functions and spherical first-kind Hankel functions, respectively. Here, ‘^′^’ indicates the first derivative. $x_{m}=\omega r\sqrt{\epsilon _{m}}/c$ and $x_{np}=\omega r\sqrt{\epsilon _{np}}/c$ are the size parameters of the matrix and the nanoparticles, respectively; $\epsilon _{np}$ is the dielectric function of nanoparticles. Here, the dielectric functions of Ag and PE are taken from Refs.^23,36^, respectively. Insulating VO_2_ is anisotropic, and its dielectric function is $\epsilon _{O}$ in the ordinary mode when it is perpendicular to the optical axis in the $x-y$ plane. The dielectric function, $\epsilon _{E}$, is in extraordinary mode along the optical axis. Using the classical oscillator formula, $\epsilon (\omega )=\epsilon _{\infty }+\sum _{i=1}^{N} \frac{S_{i} \omega _{i}^{2}}{\omega _{i}^{2}-j \gamma _{i} \omega -\omega ^{2}}$ $\epsilon _{O}$ and $\epsilon _{E}$ can be obtained. Values of high-frequency constant, $\epsilon _{\infty }$, phonon frequency, $\omega _i$, scattering rate, $\gamma _{i}$ and oscillator strength $S_i$ are taken from ref.^37^. In the metallic state, VO_2_ is isotropic and Drude model^37^ is employed to describe the dielectric function, i.e., $\epsilon (\omega )=\frac{-\omega _{p}^{2} \epsilon _{\infty }}{\omega ^{2}-j \omega \Gamma }$. To confirm the accuracy of our computational code, we performed emittance spectrum calculations for a photonic structure as described in Ghanekar’s study^38^. These results were then juxtaposed with the spectra provided in their research (see Fig. S1). The congruence of our findings with the extracted spectra from their work substantiates the reliability of our computational approach.

### Optical response of the bilateral photonic metamaterials

The absorptance (or emittance) spectra of the proposed metastructure in the metallic and insulating states are illustrated in Fig. 4a. These spectra are presented relative to the normalized AM 1.5 spectrum, atmospheric window, and 30 °C blackbody irradiation. The metastructure demonstrates a solar absorptance of 0.93, comparable to that of a black absorber while maintaining a low thermal emittance of 0.07 over the atmospheric window. This emittance value is consistent with the optical properties of metals when VO_2_ shows the optical properties of the metallic state. The optical feature of the bottom PDMS layer is not significant in the proposed metastructure due to the small period (50 nm) of the top Ag nanograting layer, which is non-comparable with the wavelengths of interest. When VO_2_ nanoparticles are in the metallic state, they exhibit behavior similar to that of Ag, Cu, and Au nanoparticles, enabling high solar absorptance. The presence of randomly distributed VO_2_ nanoparticles serves as an effective light trapper, leading to multiple reflections and scattering. This phenomenon enhances the solar reflectance of the metastructure to 0.93, even with a small volume fraction of VO_2_ nanoparticles. On the other hand, when VO_2_ is in the insulating state, its optical properties align with those of semiconductors and exhibit slight transparency over certain wavelengths. Consequently, the metastructure displays a solar absorptance of 0.2, as the Ag nanograting layer at the bottom reflects most of the incident sunlight. The PE layer, with broadband transparency from 0.3 to 25 μm, can be considered as a layer of air acting as a substrate for the VO_2_ nanoparticles. The absorption peak at 13 μm in the metastructure can be attributed to the absorption peak of VO_2_ in its insulating state. The low thermal emittance over the atmospheric window enables efficient thermal retention during the cold nighttime of winter through radiative heat transfer.

**Figure 5:**
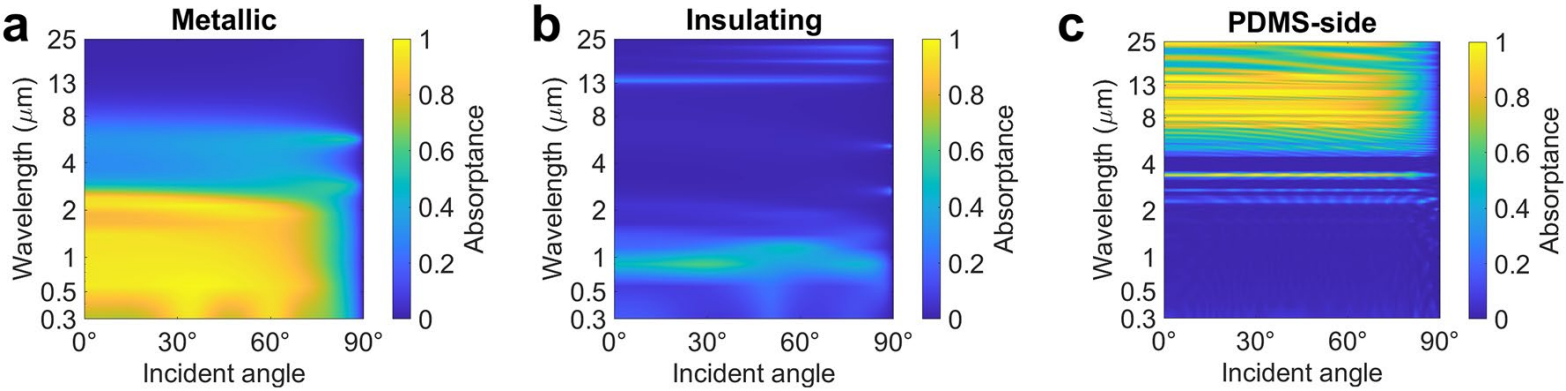
Angle- and wavelength-dependent spectra of the photonic metamaterials. Contour plot of the emittance for the bilateral metastructure varying with both wavelengths and incident angles when VO_2_ is in (**a**) metallic, (**b**) insulating states, and PDMS-side facing to the top, respectively.

The emittance spectrum of the bilateral metastructure is displayed in Fig. 4b when the PDMS side faces the top. The Ag nanograting layer is opaque and blocks the optical feature of PE nanogratings with VO_2_ nanoparticles. Therefore, the spectrum is the same whenever VO_2_ nanoparticles are in either metallic or insulating states. PDMS thin film with a reflective Ag layer is a perfect radiative cooler with a low solar absorptance of 0.04 and the molecular vibration of chemical bonds in PDMS induces a high thermal emittance of 0.9. The low solar absorptance results from the high transmittance of PDMS over solar wavelengths and the highly reflective Ag layer.

The absorptance and emittance spectra are all calculated with an incident angle of 0° while the solar irradiance varies according to seasonal and time changes. Moreover, angle-independent thermal emittance is also crucial for efficient heat dissipation over the whole hemisphere of the sky. Figure 5 exhibits the angle- and wavelength-dependent spectra of our proposed metastructure. In the metallic state, the metastructure remains high solar absorptance when the incident angle increases up to around 75° and keeps low thermal emittance from 0° to 90° as described in Fig. 5a. Moreover, the low solar absorptance and thermal emittance of the metastructure in the insulating shows a similar trend to that of the metallic state (Fig. 5b). When the PDMS side of our proposed metastructure faces the top, its low solar absorptance of 0.04 remains up to an incident angle of 80°, and its high thermal emittance of 0.9 maintains up to 75°. This feature enables efficient passive radiative cooling by reflecting sunlight from most angles of incidence and emitting thermal energy to most angles of the hemispherical sky. Next, we will build a thermal performance model to evaluate the temperature response of this metastructure.

The variation in the volume fraction of VO_2_ nanoparticles critically influences the solar absorptance across different wavelengths, as illustrated in Fig. S4. This figure demonstrates the absorptance spectra for a bilayer structure, highlighting changes as the VO_2_ nanoparticles’ volume fraction is adjusted from 5% to 20%. Notably, a significant increase in solar absorptance is observed in the range of 0.3 μm to 0.8 μm when the VO_2_ volume fraction is altered from 5% to 10%. However, as the fraction increases further from 10% to 20%, the total solar absorptance across the entire spectrum (0.3 μm to 2.5 μm) remains largely unaffected. Consequently, in our manuscript, we have chosen a 10% volume fraction for the VO_2_ nanoparticles, considering both the absorptance efficiency and the fabrication cost of the bilayer structure. Moreover, the maximum volume fraction for Maxwell-Garnet effective medium theory is 1/3, as described in this article^39^. Therefore, our calculation in this manuscript is reasonable.

### Energy balance analysis of the bilateral photonic metamaterials

In order to analyze the temperature response of the dynamic metastructure, the thermal balance equation is listed as below^22,40^:

7 $$\begin{aligned} \begin{aligned} Q_{\text{ total }}\left( T_{\text{ metastructure }}, T_{\text{ amb }}\right) =Q_{\text{ sun }}\left( T_{\text{ metastructure }}\right) +Q_{\text{ amb }}\left( T_{\text{ amb }}\right) \\ -Q_{\text{ re-emit }}\left( T_{\text{ metastructure }}\right) -Q_{\text{ non-radia }}\left( T_{\text{ metastructure }}, T_{\text{ amb }}\right) \end{aligned} \end{aligned}$$

In this situation, the back of the metastructure is thermally insulated, which means that our proposed structures are not subjected to any thermal load. Different thermal loads can cause varying temperature responses. Energy transfer takes place among the Sun, outer space, the metastructure, and the ambient environment. In this context, we use the terms $Q_{sun}$ to represent the solar heat gain, $Q_{amb}$ to describe thermal radiation from the atmosphere, $Q_{re-emit}$ to indicate the heat flux reemitted by the metastructure, and $Q_{total}$ to denote the net flux of the metastructure. Furthermore, the thermal conduction and convection heat transfer between the metastructure and the ambient is referred as to $Q_{non-radia}$.

The solar heating flux, $Q_{sun}(T_{metastructure})$, is defined by:

8 $$\begin{aligned} Q_{\text{ sun }}\left( T_{\text{ metastructure }}\right) =A \cdot C F \int _{0}^{\infty } \textrm{d} \lambda I_{\textrm{AM} 1.5}(\lambda ) \alpha \left( \lambda , \theta _{\text{ sun }}, T_{\text{ metastructure }}\right) \end{aligned}$$

**Figure 6:**
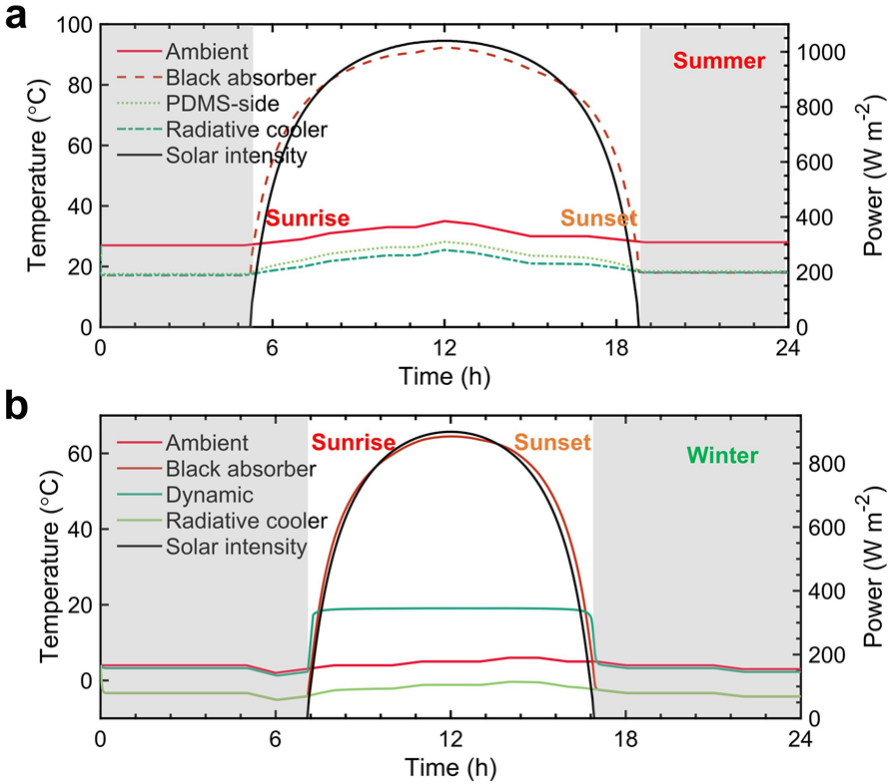
Thermal performance of the photonic metamaterials. Temperature response during the one-day cycle of the black absorber (solid black line), the ideal radiative cooler (solid green line), and the bilateral metastructure (solid light green lines) (**a**) when the PDMS side faces the top and (**b**) when the nanogratings layer faces the top..

In this context, *A* represents the area of the metastructure. The term *CF* denotes the concentration factors. $\alpha \left( \lambda , \theta _{\text{ sun }},T_{\textrm{metastructure}}\right)$ refers to the solar absorptance and it changes with temperature, wavelength, and angle of incidence. However, as shown in subsequent sections, the solar absorptance is independent of temperature and angle of incidence. Hence, we can calculate the solar absorptance when an incident angle is zero.

The power of incident thermal radiation from the atmosphere, $Q_{sun}(T_{metastructure})$ is listed as follows:

9 $$\begin{aligned} \begin{aligned} Q_{\textrm{amb}}\left( T_{\textrm{amb}}\right) =A \int _{0}^{\infty } \textrm{d} \lambda I_{\textrm{BB}}\left( T_{\textrm{amb}}, \lambda \right) \alpha \left( \lambda , \theta , \phi , T_{\textrm{metastructure}}\right) \\ \epsilon (\lambda , \theta , \phi ) \end{aligned} \end{aligned}$$

$I_{\textrm{BB}}\left( T_{\textrm{amb}}, \lambda \right) =2 h c^{5} \lambda ^{-5} \exp \left( h c / \lambda k_{B} T_{amb}-1\right) ^{-1}$ represents the thermal infrared radiation of a blackbody, where *h* denotes Planck’s constant and $k_B$ denotes the Boltzmann constant. $\alpha \left( \lambda , \theta , \phi , T_{\textrm{metastructure}}\right) =\frac{1}{\pi } \int _{0}^{2 \pi } \textrm{d} \phi \int _{0}^{\pi / 2} \epsilon _{\lambda }$ $\cos \theta \sin \theta \textrm{d} \theta$ denotes the temperature-dependent solar absorptance^41^, which is assumed to be temperature-independent based on the thermal stability test. The air emittance, $\epsilon (\lambda , \theta , \phi )$, is defined by $1-t(\lambda , \theta , \phi ). t(\lambda , \theta , \phi )$, where $t(\lambda , \theta , \phi )$ defines the transmittance of the atmosphere that is extracted from MODTRAN 4^42^.

The heat flux reemitted by the metastructure can be calculated as follows:

10 $$\begin{aligned} \begin{aligned} Q_{\text{ re-emit }}\left( T_{\textrm{metastructure}}\right) =A \int _{0}^{\infty } \textrm{d} \lambda I_{\textrm{BB}}\left( T_{\textrm{metastructure}}, \lambda \right) \\ \epsilon \left( \lambda , \theta , \phi , T_{\textrm{metastructure}}\right) \end{aligned} \end{aligned}$$

As described by the Kirchhoff’s law of thermal radiation^43^, $\epsilon \left( \lambda , \theta , \phi , T_{\textrm{metastructure}}\right)$ is equal to $\alpha \left( \lambda , \theta , \phi , T_{\textrm{metastructure}}\right)$, which represents the emittance of the metastructure.

11 $$\begin{aligned} Q_{\text{ non-radia }}\left( T_{\text{ metastructure }}, T_{\text{ amb }}\right) =h \left( T_{\text{ metastructure }} - T_{\text{ amb }}\right) \end{aligned}$$

*h* represents the thermal conduction and convection heat transfer coefficient. Here, *h* = 8 W m^-2^ K^-1^ is taken to calculate the convection heat transfer coefficient of natural air.

The change in temperature can be calculated using the following equation:

12 $$\begin{aligned} C_{\textrm{metastructure}} \frac{d T}{d t}=Q_{\textrm{total}}\left( T_{\textrm{metastructure}}, T_{\textrm{amb}}\right) \end{aligned}$$

The metastructure has a thickness of approximately 30 μm. For the purpose of calculating the temperature response, the heat capacitance of the metastructure, denoted as $C_{metastructure}$, is assumed to be equivalent to that of a 30 μm thick PDMS film, since the thickness of the Ag and PE nanograting layer is negligible in comparison and is thus ignored. The substrate thickness primarily influences the thermal load, which in turn affects the time required to reach thermal equilibrium, as demonstrated in Fig. S3.

### Thermal performance of the bilateral photonic metamaterials

In order to show how the temperature of the metastructure can be adjusted by changing its structures, we conducted simulations of the temperature response to variations in ambient temperature during the summer and winter (Fig. 6). The historical ambient temperature (solid red line in Fig. 6a) of Shanghai (Jun 29, 2022) and its solar irradiance are employed as the input data for the thermal performance evaluation. The concentration factor in Eq. 8 is not considered since we used the geographical location and the movement of the sun to extract the solar irradiance variation for a specific day from the website of PVEducation. For the summer weather, the black absorber with an approaching one solar absorptance while a thermal emittance like the commercial shingle cover and the ideal radiative cooler with spectral selectivity as depicted in Fig. 1 are employed as reference. It is shown that the black absorber reaches up to 90 °C during noontime, which demonstrates the necessity of employing passive radiative cooling materials (solid black line in Fig. 6a). The PDMS side of our proposed metastructure (solid light green line) shows a similar subambient temperature drop of about 9.5 °C during the nighttime as that of the ideal radiative cooler (solid green line) because their thermal emittance difference is only 0.1. The temperature of the metastructure is 2.7 °C higher than that of the ideal radiative cooler during the noontime. This is because the solar reflectance of the bilateral metastructure is 0.05 higher compared to the ideal radiative cooler. Consequently, the net cooling power of the metastructure is approximately 50 W m^-2^ lower than that of the ideal radiative cooler when the solar irradiance power is fixed at 1000 W m^-2^. This clearly demonstrates the cooling advantage of the bilateral metastructure, highlighting its potential for effective cooling. It is worth noting that, due to the scaling of the y-axis, the temperature trajectories for both the black absorber and radiative cooler appear identical in Fig. 6. The difference between these two curves can be seen in Fig. S2.

For the winter weather conditions, the ambient temperature and solar illumination date of Shanghai (Dec 01, 2022) are used as input data. Figure 6b shows the temperature change of the black absorber, dynamical metastructure, and ideal radiative cooler. During the nighttime, both the black absorber and the static radiative cooler exhibit a temperature that is 7.2 °C lower than the ambient temperature. This clearly highlights the drawback of the static radiative cooler. The static radiative cooler, along with the regular blackbody solar absorber, actually increases the energy consumption for space heating. The energy-saving potential resulting from the solar heating effects of the black absorber is partially offset by its radiative cooling disadvantage during nighttime. However, the nighttime temperature of the bilateral dynamic metastructure is only 0.8 °C below the ambient, which significantly reduces the space heating energy need. Furthermore, its temperature stabilizes around 20 °C which is around the critical temperature of VO_2_. This demonstrates the unique feature of the dynamical temperature regulation potential of our proposed metastructure. By varying the doping element and ratio of metals, the critical temperature of the VO_2_ can be tuned according to different application scenarios. To manufacture our proposed structure, the initial step involves the creation of the PDMS layer. This can be achieved through a roll-to-roll blade-coating process, which applies a PDMS solution and is subsequently followed by a 2-hour thermal annealing step to solidify the PDMS layer. For the deposition of the Ag layer, various techniques such as thermal vacuum evaporation, electroplating, or E-beam physical deposition can be employed. The production of VO_2_ is accomplished using the rapid hydrothermal method as described in^44^. As for the film’s matrix, the blown extrusion process commonly utilized in polymer film manufacturing can be extended for producing our proposed structure. This entails film-blown processing, where the premixed VO_2_ and PE solution are processed together, drawing from established techniques^45^. The grating structure can be done nanoprinting process.

## Conclusion

In summary, a bilateral metamaterial with dynamic and passive thermal regulation has been successfully demonstrated. The metamaterial is composed of phase-transition VO_2_, infrared-transparent PE, and infrared-absorptive PDMS. This innovative structure can automatically switch between high and low solar absorptance by taking advantage of the phase transition of VO_2_ nanoparticles at a critical temperature when the nanogratings layer faces the top. The Ag grating layer exhibits high opacity and reflectivity for both solar and infrared wavelengths. The PDMS thin film layer, in combination with the Ag grating layer, functions as an excellent infrared emitter, enabling efficient radiative cooling. The metastructure achieves high solar reflectance when the PDMS side is facing the top, due to the high solar transmittance of the PDMS thin film. The bilateral design of the metastructure confirms the feasibility and effectiveness of dynamic passive temperature regulation. Thermal simulation results demonstrate a subambient temperature drop of 6 °C during summer noontime, showcasing the radiative cooling effect. In contrast, during the cold winter daytime, the metastructure achieves dynamic solar heating regulation, maintaining a stabilized temperature of 22 °C. The integration of solar heating and radiative cooling in this advanced metamaterial opens up diverse applications across multiple sectors. It can revolutionize climate control in agricultural greenhouses, enhance comfort in portable shelters and emergency housing, and offer personalized temperature regulation in wearable technology. Its potential extends to space exploration, aiding in maintaining habitable environments in spacecraft or extraterrestrial habitats. In the realm of consumer electronics, it could improve heat dissipation in devices like laptops and smartphones. The material is also ideal for the stable transport and storage of temperature-sensitive goods, such as pharmaceuticals and perishable foods. Additionally, its application in outdoor equipment, thermal energy storage systems, architectural materials like windows, and military gear can significantly contribute to energy efficiency and sustainability in various environmental conditions.

### Supplementary Information

Supplementary Tables.

## Data Availability

The datasets used and/or analysed during the current study available from the corresponding author on reasonable request.
