# Supplementary material for: Bilateral passive thermal management for dynamical temperature regulation
Source: Sci Rep. 2024 Feb 4;14:2875. doi: 10.1038/s41598-024-53433-1 (PMC10838943; doi:10.1038/s41598-024-53433-1)
Supplement: Supplementary file 1 — Supplementary Tables. [file 41598_2024_53433_MOESM1_ESM.pdf]

# Supporting Information
# Bilateral passive thermal management for dynamical temperature regulation

**Bingyao Li**[1,2] **and Sicheng Zeng**[3,*]

[1]Shanghai University of Finance and Economics, Yangpu, Shanghai, 200433 China

[2]Shanghai Business School, Fengxian, Shanghai, 200235 China

[3]Department of Material Science and Engineering, Southern University of Science and Technology, Nanshan, Shenzhen, 518055 China

[*]Corresponding author. Email: y.zheng@northeastern.edu (Y.Z.)

**Supplementary figures:**

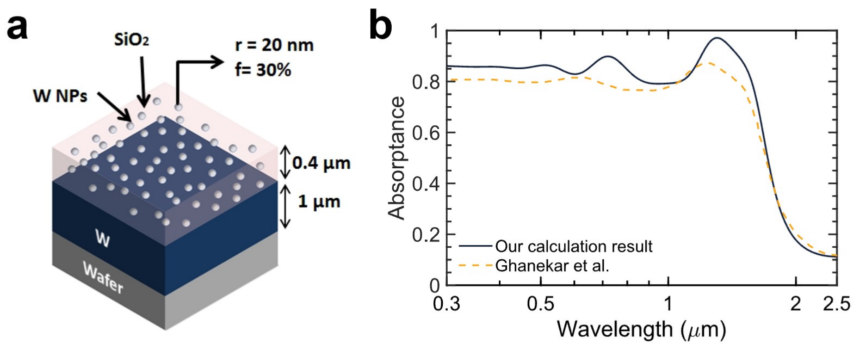


**Figure S1** (a) A design of thermal emitter consists of 0.4 $\mu$m thick $SiO_2$ layer on the top of 1 $\mu$m thick W layer deposited on the substrate. $SiO_2$ layer is doped with W nanoparticles of 20 nm radius with a volume fraction of 30%.[1] This structure is used to verify our calculation code. (b) Emittance spectra comparison of the thermal emitter in (a) using our developed code and the extracted data from Ghanekar et al's work.[1]

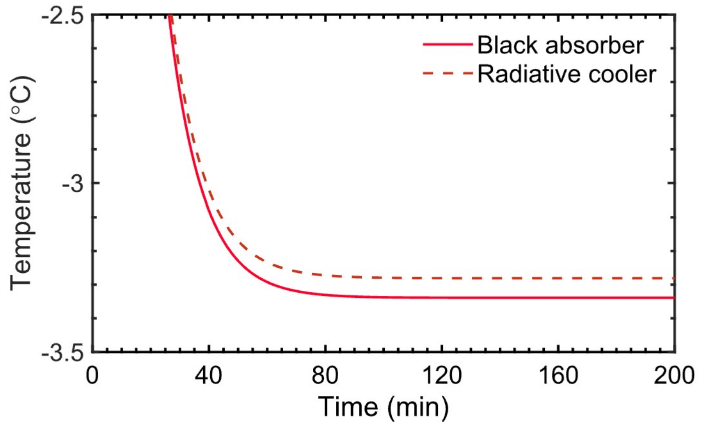


**Figure S2** Zoom-in temperature respond of black absorber and radiative cooler from 0 min to 200 min.

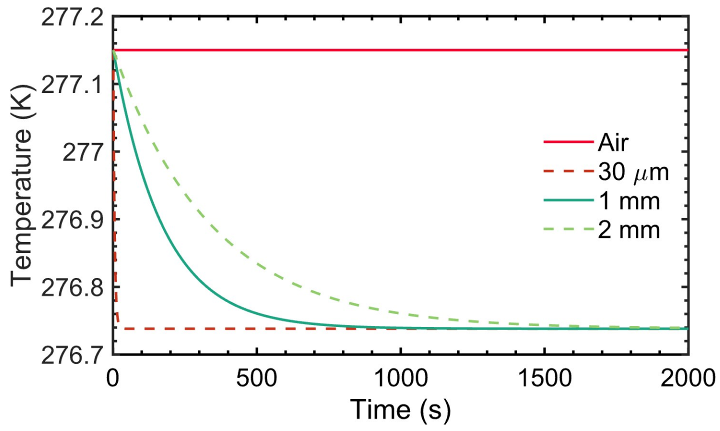


**Figure S3** The temperature response of bilayer structure when it is a free-standing film or attached on 1 mm- or 2 mm-thick plates.

We have analyzed the thermal response of a bilayer structure, considering scenarios where it is either a free-standing film or mounted on substrates of varying thicknesses (1 mm and 2 mm), as shown in Figure S3. The substrate thickness primarily influences the thermal load, which in turn affects the time required to reach thermal equilibrium, as demonstrated in Figure S3. A noticeable difference is observed when the thickness is a mere 30 $\mu$m, allowing for rapid attainment of thermal equilibrium. This duration, however, extends with an increase in substrate thickness, evident when comparing 1 mm and 2 mm thicknesses.

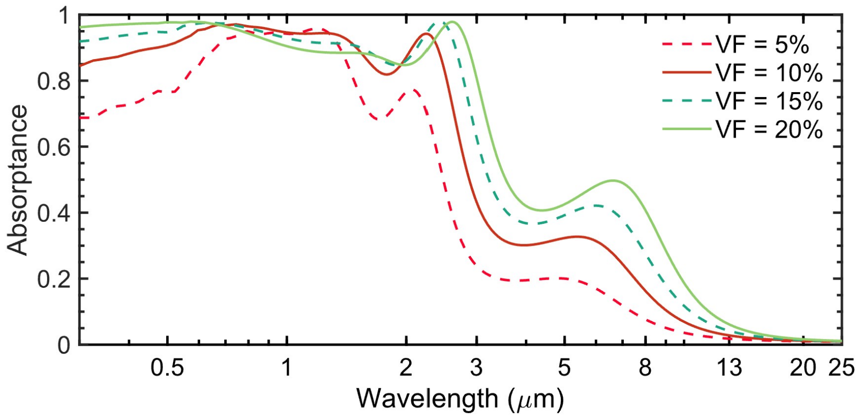


**Figure S4** Absorptance of the bilayer structure with different volume fractions of $VO_2$ nanoparticles in its metallic states.

## References

[1] Ghanekar, A., Lin, L. & Zheng, Y. Novel and efficient mie-metamaterial thermal emitter for thermophotovoltaic systems. *Optics express* **24**, A868–A877 (2016).
